# Quality of life increases in patients with painful diabetic neuropathy following treatment with spinal cord stimulation

**DOI:** 10.1007/s11136-015-1211-4

**Published:** 2015-12-22

**Authors:** Rui V. Duarte, Lazaros Andronis, Mathieu W. P. M. Lenders, Cecile C. de Vos

**Affiliations:** Department of Public Health, Epidemiology and Biostatistics, Room 124, Murray Learning Centre, University of Birmingham, Birmingham, B15 2TT UK; Department of Health Economics, University of Birmingham, Birmingham, UK; Department of Neurosurgery, Medisch Spectrum Twente, Enschede, The Netherlands; Department of Clinical Neurophysiology, University of Twente, Enschede, The Netherlands

**Keywords:** EuroQoL-5D, Neuropathic pain, Painful diabetic neuropathy, Quality of life, Spinal cord stimulation

## Abstract

**Purpose:**

This study aims to explore the changes in pain intensity and quality of life (QoL) experienced by patients with painful diabetic neuropathy (PDN) treated with spinal cord stimulation (SCS) and conventional medical practice (CMP).

**Methods:**

Patient-reported pain intensity and QoL data were obtained from participants in an international multicentre randomised controlled trial comparing SCS versus CMP. Data were collected at randomisation and 6 month follow up by means of a visual analogue scale for pain intensity, the EuroQoL Visual Analogue Scale (EQ VAS) and the EuroQol EQ-5D index. Quality-adjusted life years (QALYs) were calculated for each treatment using the ‘area under the curve’ method. Differences in QALYs were calculated after adjusting for between-treatment imbalances in baseline QoL.

**Results:**

At 6 months, patients allocated to SCS reported larger reductions in pain intensity and improvements in QoL measured by the EQ-5D utility score and EQ VAS as compared to those allocated to CMP. Initial calculations of QALYs for the SCS and CMP groups suggested no statistical differences between the groups. Adjusting for imbalances in baseline EQ-5D scores showed SCS to be associated with significantly higher QALYs compared to CMP.

**Conclusions:**

SCS resulted in significant improvement in pain intensity and QoL in patients with PDN, offering further support for SCS as an effective treatment for patients suffering from PDN. From a methodological point of view, different results would have been obtained if QALY calculations were not adjusted for baseline EQ-5D scores, highlighting the need to account for imbalances in baseline QoL.

## Introduction

Diabetes mellitus is a common chronic condition with an increasing prevalence estimated to reach 4.4 % of the world population in 2030, the equivalent of 366 million people [1]. As a result of the condition, approximately one in every three diabetic patients is expected to develop painful diabetic neuropathy (PDN) [2, 3]. PDN is defined as pain arising as a result of abnormalities in the peripheral somatosensory system in people with diabetes [4], and it is considered the most disabling and costly complication of diabetes. Several studies have reported that patients with neuropathic pain experience lower levels of health-related quality of life (QoL) when compared to the general population [5, 6]. More specifically, PDN may interfere substantially with QoL aspects such as general activity, mood, mobility, self-care, recreational and social activities [7].

A number of oral pharmacologic treatment options are available for the management of PDN, including serotonin-norepinephrine reuptake inhibitors (SNRIs), tricyclic antidepressants, carbamazepine, venlafaxine, duloxetine, and amitriptyline. These may be effective for short-term management of PDN [8]. However, the majority of diabetic patients report persistent pain over several years even following pharmacologic treatment [9].

Spinal cord stimulation (SCS) is a widely-used intervention for the management of neuropathic pain conditions, and it has been suggested as a promising treatment option for PDN. Randomised controlled trials (RCTs) have demonstrated the effectiveness of SCS in the management of failed back surgery [10] and complex regional pain syndrome [11]. Recently, the effectiveness of SCS for PDN was investigated in an RCT comparing SCS against conventional medical practice (CMP) [12]. The results of this study showed that patients treated with SCS presented statistically significant improvements in pain relief and QoL. However, the QoL analysis was merely based on data captured through the EuroQol visual analogue scale (EQ VAS). Another recent RCT evaluating SCS for PDN observed improvements in pain relief but not in QoL [13]. Thus, evidence on the effect of SCS on QoL of patients with PDN remains inconclusive.

We hypothesise that patients with PDN treated with SCS obtain larger reductions in pain intensity and improvements in QoL when compared to those patients with PDN treated with CMP alone. The aim of this study was to explore the changes in pain intensity and QoL experienced by patients with PDN treated with SCS and CMP. To this end, we analysed patient responses to three instruments (visual analogue scale for pain intensity (VASPI), EQ VAS, EQ-5D index) obtained from a multicentre randomised controlled trial.

## Methods

### Study design and patient recruitment

The design and results of the RCT have been described previously in detail [12]. In brief, a total of 60 patients diagnosed with PDN were recruited from seven pain clinics in the Netherlands, Denmark, Belgium and Germany between November 2008 and October 2012. Patients were eligible for inclusion if they were 18 years of age or older, were diagnosed with refractory diabetic neuropathic pain in the lower extremities for more than 1 year and had a pain intensity score of at least 50 on the 100 mm VASPI scale, which ranges from 0 (no pain) to 100 (worst possible pain) despite previous treatment with available conventional treatments. Upon recruitment, patients were stratified for gender and centre, and were randomised in a 2:1 ratio to either CMP alone (CMP group, *n* = 20) or conventional medical practice supplemented by SCS (SCS group, *n* = 40). Patients randomised to the SCS group underwent a screening trial lasting up to 7 days to assess their response to SCS, and a pulse generator was only implanted if the screening trial was successful. 6 months post randomisation, patients in the CMP group could cross over to SCS therapy if adequate improvement had not been achieved.

### Outcomes and data collection

Information on patients’ age, gender, type and duration of diabetes, pain intensity and onset of pain was collected prior to randomisation (baseline). Pain intensity was assessed using a 100 mm visual analogue scale ranging from 0 (no pain) to 100 (worst possible pain) [14]. The VASPI is considered a reliable and valid measure of subjective phenomena including chronic pain [14, 15]. Clinically important changes were determined in accordance with a consensus statement that established a 10–30 % decrease as minimal clinically important, ≥30 % as moderate clinically important and ≥50 % as a substantial clinical change [16]. Health-related quality of life was derived from participants’ responses to the EuroQoL EQ-5D instrument. This included the EQ VAS and the EQ-5D (three level) descriptive system. The EQ VAS resembles a thermometer on which respondents record their self-rated health where the lower and upper ends are labelled 'worst' and 'best' imaginable health state, respectively. The EQ-5D descriptive system is a questionnaire designed to be completed by the patient and comprising five dimensions (mobility, self-care, usual activities, pain/discomfort and depression/anxiety), where each dimension has three levels: no problems, some problems and extreme problems. The respondent is asked to indicate his/her overall health state by selecting the level that corresponds to his/her quality of life for each of the five dimensions. Responses to the EQ-5D descriptive system were converted into single (utility) indices using a set of weights (tariff) reflecting population preferences for the particular health state. In this study, utility scores were obtained by using the Dutch tariff [17]. QALYs were calculated by the area-under-the-curve (AUC), involving linear interpolation of utility indices over the study period [18].

### Statistical analysis

Comparisons of scores obtained from self-reported measures (VASPI, EQ VAS, EQ-5D) between groups were carried out using independent-samples *t* tests. Changes in these scores between different time points (baseline and 6 month follow up) were assessed using paired-samples *t* tests. Changes in levels of EQ-5D dimensions were evaluated through the Mann–Whitney test for between-group analyses, and the Wilcoxon signed-rank test for within-group analyses. Baseline EQ-5D scores are a strong predictor of total QALY scores, therefore mean differences in QALYs were calculated after adjusting for imbalances in baseline scores between groups [19]. Mean differences in QALYs between the SCS and CMP groups are presented alongside confidence intervals obtained from 5000 bootstrap replications (bias corrected and accelerated method). Sensitivity analyses were carried out using the intention-to-treat (ITT) principle and missing data imputed using first observation carried forward. The results of these analyses were not different from the results presented within this paper. In addition, we run further analyses to explore the effect of available covariates, including gender, age, duration of pain, duration and type of diabetes, baseline VASPI, EQ VAS and EQ-5D index score. We found that the only statistically significant variables were group (treatment group) and baseline EQ-5D index score (data not shown). Statistical analyses were carried out in STATA (Release 13.1; College Station, TX: StataCorp LP).

## Results

Baseline characteristics of the study sample are reported in Table 1. Recruited patients had a mean duration of diabetes of 16 years, with 75 % of them having Type II diabetes. The mean duration of pain was 7 years. The mean pain score across all participants was 72 on the VASPI, the mean EQ-5D utility score obtained from the health status classification instrument was 0.33 and the mean score obtained from the EQ VAS was 49. Three patients in the SCS group did not proceed to implantation of SCS. Two of these patients did not perceive significant pain relief and in one patient it was not possible to implant the electrode lead. One additional patient is the SCS group was withdrawn despite good response to SCS after deciding to enter a pharmacological gastroenterology study. In the CMP group, two patients withdrew consent after 3 months due to experiencing new diseases unrelated to their PDN condition. These patients (SCS = 4; CMP = 2) were not included in the 6-month follow-up analysis.

**Table 1:** Baseline characteristics

	All participants (*n* = 60)	CMP group (*n* = 20)	SCS group (*n* = 40)
Male, *n* (%)	38 (63)	13 (65)	25 (63)
Age in years, mean (SD)	59 (11)	61 (12)	58 (11)
Type I diabetes, *n* (%)	15 (25)	5 (25)	10 (25)
Type II diabetes, *n* (%)	45 (75)	15 (75)	30 (75)
Duration of diabetes in years, mean (SD)	16 (12)	17 (12)	15 (11)
Duration of pain in years, mean (SD)	7 (5)	7 (6)	7 (6)
Pain VASPI, mean (SD)	72 (15)	67 (18)	73 (16)
EQ-5D, mean (SD)	0.33 (0.29)	0.47 (0.31)	0.27 (0.26)
EQ VAS, mean (SD)	49 (18)	48 (16)	50 (19)

*CMP* conventional medical practice; *SCS* spinal cord stimulation; *VASPI* visual analogue scale for pain intensity

In the SCS group, minimal clinically important reductions in pain intensity (10–30 %) were reported by four (11 %) of the patients, moderate important reductions (30–50 %) were experienced by three (8 %) while substantial clinical differences (≥50 %) were reported by 24 (67 %) of the patients. Of the patients randomised to CMP, six (33 %) reported minimal clinically important reduction in pain intensity and only one (6 %) patient reported ≥50 % pain relief.

**Fig. 1:**
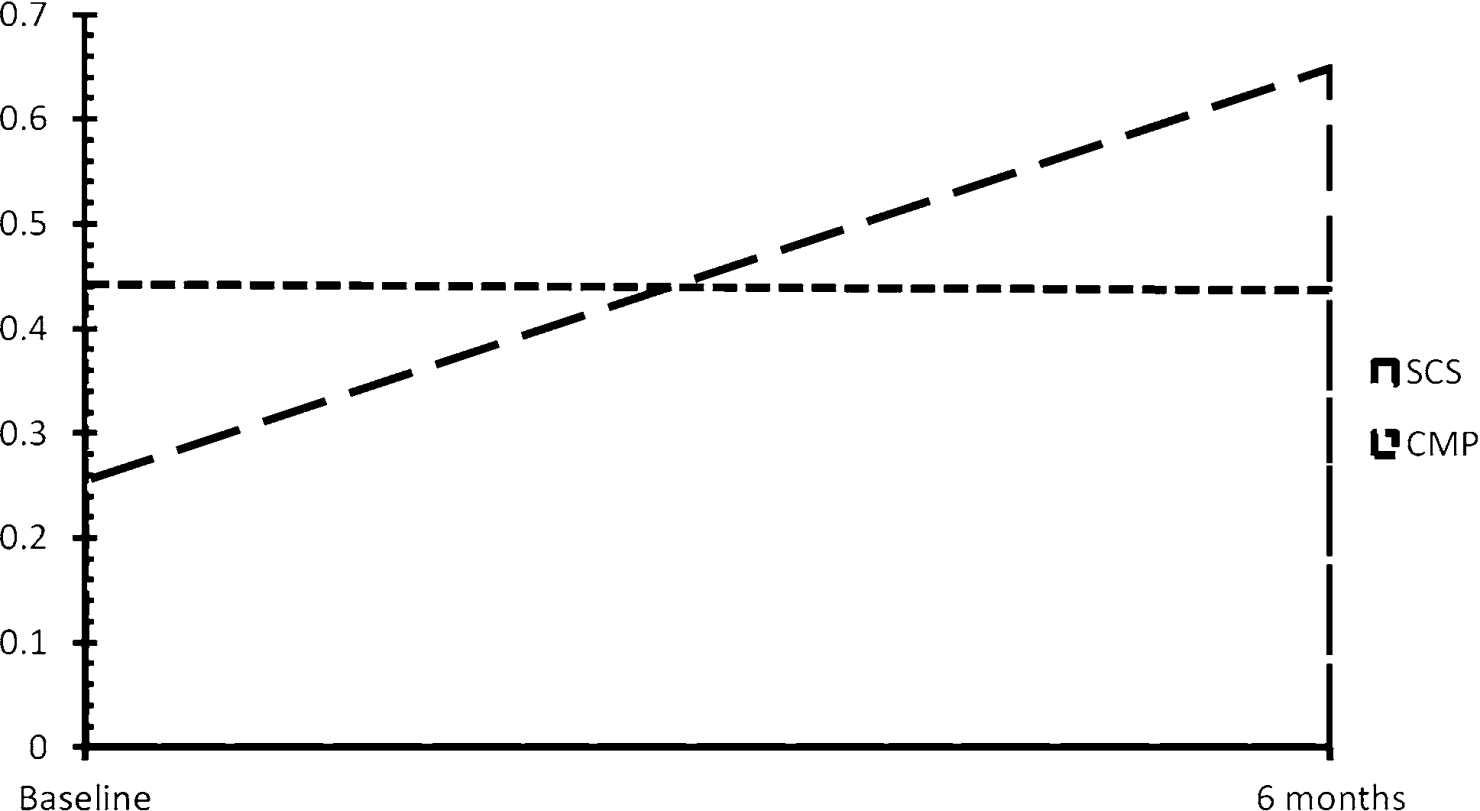
EQ-5D index scores for SCS and CMP at baseline and 6 months follow-up. *CMP* conventional medical practice; *SCS* spinal cord stimulation

No statistically significant differences were observed for the CMP group between baseline and 6-month follow-up for the VASPI, EQ-5D utility or EQ VAS scores (Table 2). Statistically significant improvements were observed for all outcome measures for the patients in the SCS group between baseline and 6-month follow-up. Patients randomised to SCS experience greater pain relief and greater improvement in QoL as measured by the EQ-5D utility scores and EQ VAS than those patients randomised to CMP. However, the EQ-5D utility scores at baseline were statistically significantly different between SCS and CMP groups (Fig. 1).

**Table 2:** Pain and EQ-5D scores for the SCS and CMP treatment groups

	Baseline			6 months			Within-group difference	
	SCS (*n* = 40)	CMP (*n* = 20)	SCS-CMP difference	SCS (*n* = 36)	CMP (*n* = 18)	SCS-CMP difference	SCS (*n* = 36)	CMP (*n* = 18)
	Mean (SD)	Mean (SD)	Mean (95 % CI)	Mean (SD)	Mean (SD)	Mean (95 % CI)	Mean (95 % CI)	Mean (95 % CI)
VASPI	73 (16)	67 (18)	−6 (−15 to 3)	29 (27)	66 (22)	37 (22–52)^^^	46 (35–)**	0.5 (−10 to 11)
EQ-5D index	0.27 (0.26)	0.47 (0.31)	0.20 (0.05–0.36)^	0.65 (0.28)	0.44 (0.33)	−0.21 (−0.39 to −0.04)^	−0.39 (−0.50 to −0.29)**	0.00 (−0.10 to 0.11)
EQ VAS	50 (19)	48 (16)	−1 (−11 to 8)	61 (23)	41 (20)	−20 (−34 to −7)^^	−12 (−22 to −1)*	7 (−1 to 15)

*CMP* conventional medical practice; *SCS* spinal cord stimulation; *VASPI* visual analogue scale for pain intensity

^ *p* < .05; ^^ *p* < .01; ^^^ *p* < .001 (between groups); * *p* < .05; ** *p* < .001 (within a group)

Comparison of the SCS (*M* = 0.23, SD = 0.11) and CMP (*M* = 0.22, SD = 0.15) groups based on QALYs calculated as the area under the curve did not show statistically significant differences between treatments (*p* = 0.87; 95 % CI 0.07–0.08). As baseline QoL is a strong predictor of total QALY scores, we calculated differences in QALYs between groups by adjusting for baseline imbalances in EQ-5D scores (Table 3). The results showed statistically significant differences in QALYs between the groups (*p* < 0.001; 95 % CI 0.04–0.11). Patients randomised to SCS experienced a higher QALY gain when compared to the patients receiving CMP.

**Table 3:** QALYs unadjusted and adjusted for baseline EQ-5D score over a period of 6 months per treatment group

	SCS	CMP	Difference	95 % CIs*	
QALYs—unadjusted for baseline EQ-5D score	0.226	0.220	0.006	−0.070	0.085
QALYs—adjusted for baseline EQ-5D score	0.258	0.178	0.080^^^	0.044	0.114

* 95 % non-parametric confidence intervals based on 5000 bootstrap bias corrected replicates

^^^ *p* < 0.001

**Fig. 2:**
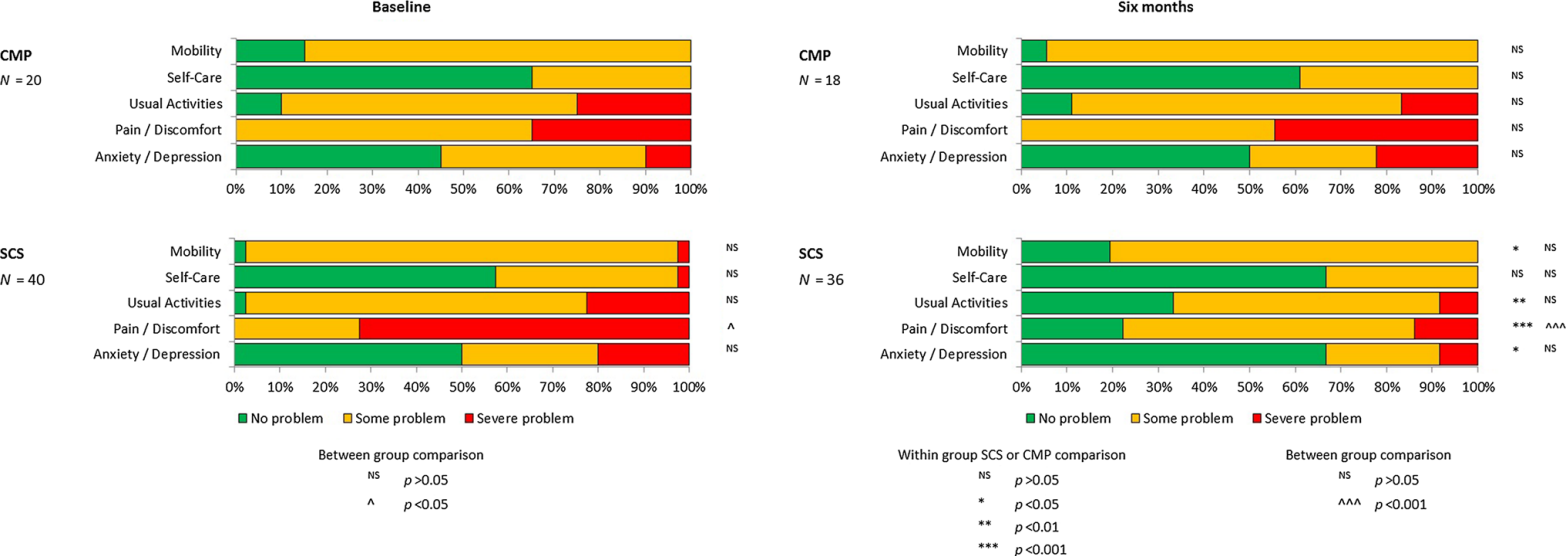
Comparison of the EQ-5D subcategories in CMP and SCS groups at baseline and 6 month follow-up. *CMP* conventional medical practice; *SCS* spinal cord stimulation

On the EQ-5D dimensions, at 6-months the patients randomised to SCS reported significant improvements in four out of five dimensions: mobility, usual activities, pain/discomfort and anxiety/depression when compared to baseline (Fig. 2). Statistically significant differences were observed between groups for the pain/discomfort dimension.

Of the 18 patients that received CMP until 6-months, 14 (78 %) crossed-over to SCS following the 6-month follow-up. Of the 36 patients that were implanted with an SCS, 34 (94 %) continued to receive SCS therapy after the 6-month study endpoint.

## Discussion

This study analysed data obtained from an international multicentre RCT to compare the levels of quality of life reported by patients treated with SCS and CMP. Results showed that SCS leads to statistical and clinical significant improvement in pain intensity and quality of life in patients with PDN as compared to CMP.

Pain intensity findings are consistent with previous non-randomised studies of SCS in patients with PDN [20–23]. These studies observed that the majority of patients receiving SCS experienced 50 % pain relief or more after 12 months.

The results of this study are based on a 6-month follow-up. This was the primary endpoint of the de Vos et al. RCT [12]. The effects of SCS in pain relief and QoL have been found to be sustained at 24-month follow-up of RCTs for other neuropathic pain conditions [24, 25]. It has been suggested that the effects of SCS for complex regional pain syndrome Type I may diminish after 2 years of treatment with no differences for pain relief and all other measured variables between SCS and physical therapy [26]. Over 50 % pain relief after 3 years of SCS therapy was reported by five out of six patients with PDN in a non-randomised study [27]. Long-term follow-up of the cohort of this RCT is required to verify if these results are corroborated in patients with PDN.

Painful diabetic neuropathy may interfere substantially with quality of life aspects [7]. Interpretation of the baseline scores of this trial indicates that the pain experienced by patients with PDN had a negative impact in their QoL interfering mainly with their usual activities and mobility. Four of the five dimensions of the EQ-5D improved significantly at 6-months following treatment with SCS. Similar results with SCS have previously been described in a patient group with failed back surgery syndrome [28]. Significant improvements in QoL were observed for the patients receiving SCS based on both the EQ VAS and EQ-5D utility scores. Recently an additional RCT evaluated SCS compared to best medical treatment in patients with PDN [13]. Statistically significant improvements following SCS were observed for pain intensity but not for QoL when analysing both the EQ VAS and the EQ-5D utility scores. The authors suggested that this may have been due to the large variability of the data and the limited number of participants. It is unclear if the analysis of the EQ-5D was adjusted to possible imbalances in baseline scores. In addition, EQ-5D index scores reported in this study were calculated based on the UK tariff although the study was conducted in the Netherlands. The reasons for choosing to use the UK tariff instead of the Netherlands one were not presented.

An initial evaluation of the QALYs based on calculation of the area under the curve suggested that there were no statistically significant differences between the groups. However, it has been argued that such results may be biased due to imbalances in baseline utility scores [19]. Further analysis indicated that the EQ-5D baseline utility scores were significantly different, with the patients randomised to CMP having greater utility levels. Five outliers in the EQ-5D baseline utility scores were identified, but these were in the SCS group and in the upper level. Therefore, excluding these outliers would only accentuate the baseline EQ-5D index scores difference between the groups. Analysis of QALYs while adjusting for the baseline utility scores resulted in statistically significant differences, demonstrating that patients randomised to SCS obtained greater QALY gains than those receiving CMP. Not taking into account potential imbalances in baseline utility scores may result in misleading interpretation of QALY results with potential implications in the cost-effectiveness evaluation of treatments.

The subjects of this study were derived from the de Vos et al. [12] trial which had a number of strengths, including a low attrition rate and a comprehensive collection of outcome measures including pain and health-related quality of life. We believe the current study is the first to provide a detailed report of the sub-categories of the EQ-5D in patients with refractory PDN of the lower extremities. Study limitations included lack of blinding, however this was not possible with the SCS device used due to the patients feeling paraesthesia in the area of the pain when the stimulation is on. We acknowledge that lack of blinding is a potential source of bias. However, due to the nature of the intervention and comparator it would not be feasible to blind patients. Recently developed paraesthesia-free devices may allow for a double-blind design, with both arms receiving a device but only one of the arms receiving active treatment.

## Conclusions

In conclusion, the pain experienced by the patients recruited for this study had a negative impact on their quality of life. When analysing QALYs based on the area under the curve, it is important to take into consideration possible baseline imbalances in EQ-5D utility scores. Disregarding baseline imbalances could potentially lead to erroneous and misleading conclusions. Spinal cord stimulation resulted in clinical and statistical improvements in pain and quality of life of patients with painful diabetic neuropathy. The results of this study further support spinal cord stimulation as an effective alternative for those patients with refractory painful diabetic neuropathy in the lower extremities.
